# Machine learning assisted breathomic approach for early-stage thoracic cancer detection

**DOI:** 10.3389/fonc.2025.1635280

**Published:** 2025-09-17

**Authors:** Zhenguang Chen, Minhua Peng, Pengnan Fan, Sai Chen, Xinxin Cheng, Bo Xu, Ruiping Chen, Xiao Hu, Wei Wei, Tingting Zhao, Jun Kong, Weiliang Liang, Xiangcheng Qiu, Sitong Chen, Junqi Wang

**Affiliations:** ^1^ Department of Thoracic Surgery, The First Affiliated Hospital, Sun Yat-sen University, Guangzhou, Guangdong, China; ^2^ ChromX Health Co., Ltd., Guangzhou, Guangdong, China; ^3^ Center for Private Medical Service & Healthcare, The First Affiliated Hospital, Sun Yat-sen University, Guangzhou, Guangdong, China; ^4^ State Key Laboratory of Oncology in South China, Sun Yat-sen University Cancer Center, Guangzhou, Guangdong, China; ^5^ Department of Thoracic Surgery, Guizhou Hospital of the First Affiliated Hospital of Sun Yat-sen University, Guiyang, Guizhou, China; ^6^ Jingjinji National Center of Technology Innovation, Beijing, China

**Keywords:** breathomics, volatile organic compounds, exhaled breath, thoracic cancer, machine learning, early diagnosis, thermal desorption-gas chromatography-mass spectrometry, postoperative monitoring

## Abstract

**Objective:**

This study explores the feasibility of using breathomic biomarkers analyzed by machine learning as a non-invasive diagnostic tool to differentiate between benign and malignant thoracic lesions, aiming to enhance early detection of thoracic cancers and inform clinical decision-making.

**Methods:**

This study enrolled 132 participants with confirmed diagnosis of lung cancer, esophageal cancer, thymoma, and benign diseases. Exhaled breath samples were analyzed by thermal desorption-gas chromatography-mass spectrometry. A logistic regression algorithm was employed to construct a classification model for benign and malignant thoracic lesions. This model was trained on a subset of 80 cases and subsequently validated in a separate set comprising 52 samples.

**Results:**

A logistic regression model based on thirteen exhaled volatile organic compounds (VOCs) was developed to differentiate benign and malignant thoracic lesions. The 13-VOC model achieved an AUC of 0.85 (0.72, 0.96), accuracy of 0.79 (0.66, 0.88), sensitivity of 0.82 (0.67, 0.91), and a specificity of 0.71 (0.45, 0.88). It correctly classified 80% of lung cancer, 80% of thymoma, and 100% of esophageal cancer cases, distinguishing 71.4% of benign lesions. For lung cancer, the model achieved an AUC of 0.79 (0.57, 0.98), sensitivity of 0.80 (0.63, 0.91), and specificity of 0.63 (0.31, 0.86), with 81.8% accuracy in detecting early-stage (Stage 0 + I + II) disease. The model outperformed a 4-serum tumor marker panel in sensitivity (0.90 vs. 0.39, *p* < 0.001). Additionally, in a cohort of 58 cancer patients, model-predicted risk significantly decreased post-surgery (*p* < 0.01), indicating a strong correlation with disease burden reduction.

**Conclusion:**

This study demonstrates the feasibility of utilizing breathomics biomarkers for developing a non-invasive machine learning model for the early diagnosis of thoracic malignancies. These findings provide a foundation for breath analysis as a promising tool for early cancer detection, potentially facilitating improved clinical decision-making and enhancing patient outcomes.

## Graphical Abstract

This ML-based breathomics study employs TD-GC-MS to analyze 13 exhaled VOCs, achieving superior sensitivity to 4 serum markers in distinguishing lung/thymoma/esophageal cancers from benign conditions. Post-surgical risk reduction confirmed VOC biomarkers' correlation with disease burden.

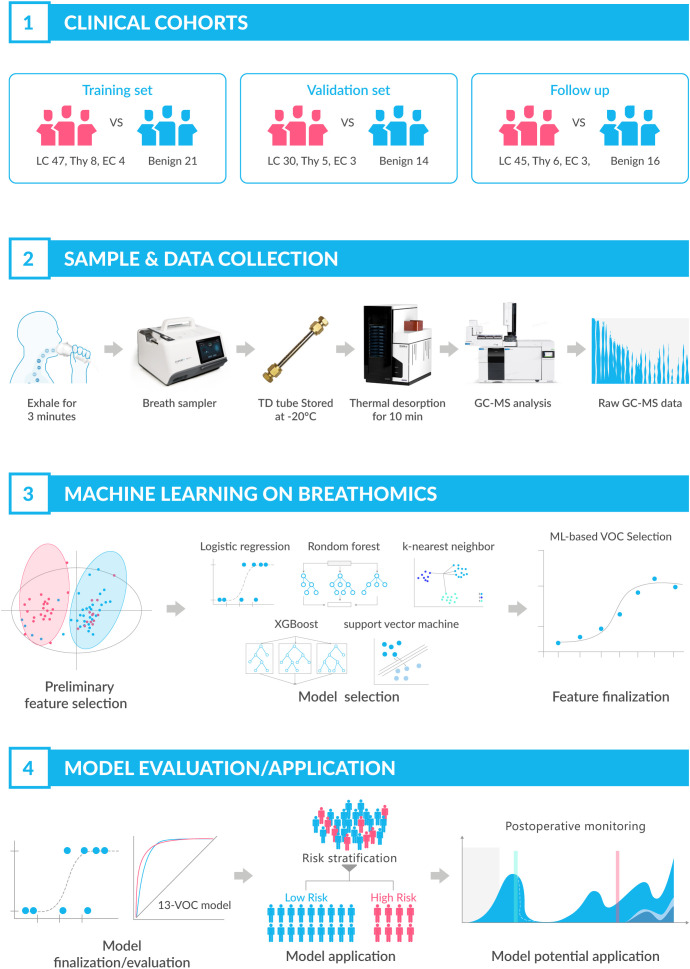

## Introduction

Thoracic malignancies, particularly lung and esophageal cancers, represent a significant global health burden. Lung cancer is the leading cause of cancer-related deaths worldwide, with nearly 2.5 million new cases and over 1.8 million fatalities in 2022 (1). Despite treatment advancements, the five-year survival rate remains below 20%, primarily due to diagnoses at advanced stages (2). Similarly, the prognosis for esophageal cancer is also bleak, with a five-year survival rate below 20% (3), mirroring the situation for lung cancer, as evidenced by 511,000 new cases and 445,000 deaths attributed to the disease worldwide in 2022 (1). Thymomas, though rare, can lead to serious complications like myasthenia gravis. While their global incidence is between 0.13 and 0.26 per 100,000 individuals (4), their impact on patients’ quality of life is significant. These factors underscore the urgent need for improved diagnostic modalities for thoracic malignancies. Current diagnostic approaches, including imaging and invasive procedures like biopsy, the gold standard for thoracic tumor diagnosis, face limitations such as the imprecision to reliably differentiate benign from malignant lesions and the risk of complications associated with invasive procedures (5, 6). Particularly, the limited sensitivity of conventional blood-based tumor marker assays is underscored by the fact that 60-70% of cases are diagnosed at late stages, primarily due to these methods’ inability to detect early biological changes​ and distinguish between overlapping clinical features, thereby delaying timely intervention (4, 7, 8). Therefore, there is an urgent need to develop more precise, non-invasive, and highly sensitive tools to improve the early detection and diagnostic accuracy of thoracic tumors.

Exhaled breath volatile organic compounds (VOCs) are carbon-based molecules, primarily derived from endogenous metabolic processes and systemic circulation. Over 3,000 VOCs have been identified, reflecting the complex metabolic activity within the human body (9–12). Disease processes (e.g., oxidative stress, inflammation) or pathogens (e.g., bacteria and viruses), can perturb normal metabolic pathways, including lipid peroxidation, amino acid metabolism, and carbohydrate metabolism, leading to unique alterations in the VOC profile, creating disease-specific signatures (13). These VOCs, diffusing from blood into breath, serve as dynamic biomarkers, enabling the detection of subtle changes associated with disease onset and progression. Breath analysis of these VOC profiles thus offers a non-invasive, real-time method for early disease detection (14). Previous studies have demonstrated the potential of breath VOCs as biomarkers in identification of a variety of cancers, including lung cancer (15–18), breast cancer (19), and gastrointestinal malignancies (20–22). Gordon et al. were pioneers in using gas chromatography-mass spectrometry (GC-MS) to identify alkenes in the breath of lung cancer patients (23). Kumar et al. reported that a panel of 12 VOCs detected using a profile-3 selected ion flow tube mass spectrometry instrument could distinguish esophageal cancer from normal controls, achieving an AUC of 0.97 in the initial analysis and 0.92 ± 0.01 in the validation set (24). However, research efforts have predominantly focused on distinguishing cancer patients from healthy controls, with limited emphasis on differentiating between benign diseases and cancer patients. Furthermore, to the best of our knowledge, no studies have investigated the use of VOCs as biomarkers for identifying thymomas so far. This distinction is particularly important in thoracic tumors, where benign diseases such as granulomas or hamartomas may mimic malignancies on medical imaging, leading to diagnostic uncertainty and potentially unnecessary invasive procedures.

This study introduces a novel machine learning model that employs a comprehensive panel of breath-derived VOC biomarkers analyzed using GC-MS to achieve simultaneous early detection of lung cancer, esophageal cancer, and thymoma—the first breathomics-based strategy for multi-thoracic cancer diagnosis. By evaluating pre/postoperative predictions, we will assess its potential for real-time postoperative monitoring. Notably, we will also compare the sensitivity of this breath-based approach with conventional blood-based tumor markers, with the goal of providing a non-invasive solution for early detection and postoperative monitoring of thoracic cancers.

## Methods

### Study design and participants

This cross-sectional study, conducted from November 2021 to January 2022 at the East Division of the First Affiliated Hospital of Sun Yat-sen University in Guangzhou, China, received approval from the Ethics Committee of the First Affiliated Hospital of Sun Yat-sen University (No. 2022-016). All subjects provided signed informed consent. Inclusion criteria were adult participants aged 18–80 years with clinical suspicion of malignant thoracic tumors, supported by imaging evidence (CT/PET-CT) and a multidisciplinary team (MDT) assessment prior to histological confirmation. Eligible participants encompassed treatment-naïve, newly diagnosed thoracic cancer patients scheduled for surgical resection for diagnostic evaluation, and patients with a history of treated or recurrent thoracic malignancy, provided comprehensive treatment records were available. Exclusion criteria encompassed individuals who were unwilling or unable to provide in-person informed consent, those with unqualified breath samples, patients with relapsed diseases and incomplete treatment histories, individuals suffering from other malignant tumors, those with severe bronchial asthma or confirmed tuberculosis, and those with severe liver damage or kidney diseases. Each participant had undergone resection surgery and was pathologically confirmed to be categorized into one of the following groups: lung cancer, thymoma, esophageal cancer, and benign disease controls. Demographic and clinical information were meticulously recorded and collected. This study was registered in the Chinese Clinical Trial Registry (Registration No.: ChiCTR2200061264).

### Exhale breath collection

All samples were collected following the same standardized procedure. Prior to collection, subjects were asked to rinse their mouths with purified water and rest for 15 minutes to stabilize their respiratory patterns. All subjects were required to abstain from food and beverages except water and smoking for at least 12 hours before the collection. To minimize the influence of diurnal metabolic variations, all collections were scheduled between 7:00 AM and 9:00 AM.

**Figure 1:**
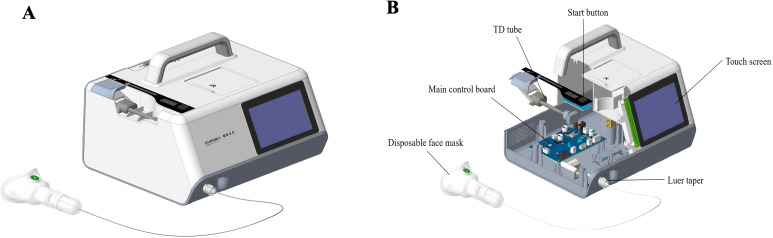
Exhaled breath collection device. The images depicted the schematic diagram **(A)** and sectional view **(B)** of the exhaled breath collection device.

Subjects were instructed to remain seated and breathe normally through a mask for 3 minutes. During exhalation, breath samples were concurrently drawn through a breath sampler (CXBC-Alpha, ChromX Health Co., Ltd) containing an internal sampling pump and a flow control module (**Figure 1**). 900 mL of breath samples were collected at a rate of 300 mL/min and directed into thermal desorption tubes. These tubes, pre-conditioned with 99.9% nitrogen gas to ensure a clean and inert environment, contained Carbopack X and Carbopack B for sample enrichment, concentrating the target compounds for later analysis. All collected samples were sealed with inert end caps immediately and stored at -20°C to maintain their integrity and analyzed by thermal desorption-gas chromatography-mass spectrometry (TD-GC-MS) within 7 days to ensure timely and accurate results.

### TD-GC-MS analysis

Breath samples were analyzed by TD-GC-MS using a system incorporating a high-throughput autosampler, a thermal desorber (TD100-xr, MARKES), and an 7890B-5977A GC/MSD (Agilent Technologies). Separation was performed on an HP-5MS capillary column with nitrogen carrier gas. The mass spectrometer operated in electron ionization (EI) mode at 70 eV, acquiring data in full scan mode (m/z 33-450). Detailed instrument parameters are provided in Supplementary Materials.

### GC-MS quantification and pre-analysis quality control

Raw GC-MS data were processed using MSDial v5.4 for peak detection, quantification, and alignment. The software generated matrices of peak area (VOC area matrix) and signal-to-noise ratio (SNR matrix). Prior to statistical analysis, a data preprocessing and filtering protocol was implemented in Python 3.9.18 to ensure data robustness. Firstly, the signal-to-noise ratio (SNR) matrix was used to assess response reliability. A VOC measurement was classified as valid if its SNR value exceeded 10; measurements below this threshold were excluded due to significant noise interference. For individual samples, the response rate was calculated as the percentage of valid VOC measurements relative to the total measurements in the sample. Samples with a response rate ≥80% were retained for further analysis. Similarly, compound-specific response rates were determined for each VOC by calculating the proportion of valid measurements across all samples. To ensure analytical robustness, only VOCs with a response rate ≥50% were included in the validated dataset, which was designated as the “valid VOC area matrix”. Secondly, the valid VOC area matrix was then log10-transformed to address heteroscedasticity, followed by normalization to account for variations in instrument response and sample loading. These steps enabled meaningful comparison of VOC abundances across samples.

### Dataset partition

A dataset of 132 participants with malignant or benign thoracic lesions was used in this study, comprising 97 malignant and 35 benign samples. For biomarker discovery and model development, the dataset was randomly split into a discovery set (60%, *n* = 79; 59 malignant, 20 benign) and a testing set (40%, *n* = 53; 38 malignant, 15 benign). The discovery set (training set) was used for feature selection and model training, while the testing set served for independent model evaluation.

### Biomarker screening

To identify VOCs differentially expressed between malignant and benign thoracic lesions, two complementary approaches were employed. First, the Wilcoxon rank-sum test was used to assess the distribution of individual VOCs across the two groups, generating corresponding *p*-values. Second, orthogonal partial least squares-discriminant analysis (OPLS-DA) was performed to evaluate the collective contribution of VOCs to group classification and to calculate variable importance in projection (VIP) scores (25). VOCs meeting both criteria of a *p* - value < 0.05 and a VIP score > 1 were selected as candidate biomarkers.

Putative biomarker identification was subsequently conducted using Agilent MassHunter Qualitative Analysis 10.0 software and the NIST 17 mass spectral library. Finally, metabolic pathway-associated VOCs reported in the literature were selected as candidate biomarkers for inclusion in diagnostic model development.

### Machine learning algorithms selection and evaluation

Given the complexity inherent in omics data, it is essential to identify the most suitable model for the dataset at hand. To this end, five commonly used machine learning algorithms were systematically evaluated: logistic regression (LR) (26), random forest (RF) (27), *k*-nearest neighbors (KNN) (28), eXtreme Gradient Boosting (XGBoost) (29), and support vector machine (SVM) (30). Among these, logistic regression algorithm demonstrated the highest robustness and effectiveness, based on its superior performance across both the discovery and testing datasets. Consequently, logistic regression model was deployed for diagnostic prediction.

### Feature selection

To minimize overfitting, a progressive feature selection approach was employed. Biomarkers were ranked by their area under the receiver operating characteristic curve (ROC-AUC) scores. A logistic regression model was trained using 5-fold cross-validation with stratified sampling, iteratively adding one feature at a time, starting with the highest-ranked biomarker. This process continued until no further significant improvement in model performance was observed.

### Hyperparameter optimization

With the optimal feature subset identified, logistic regression hyperparameters were tuned using grid search with stratified sampling. The following hyperparameters were considered: regularization method, regularization strength, early stopping criteria, and class weights. The parameter combination that yielded the highest AUC score was selected for final model training.

### Final model evaluation

The final logistic regression model, incorporating the optimized feature subset and hyperparameters, was trained on the training dataset. The model was then finalized, and a classification threshold was determined using the Youden index. Subsequently, the model’s performance was evaluated independently on the validation dataset. Performance was assessed using five metrics: F1-score, accuracy, sensitivity, specificity, and AUC, along with their respective confidence intervals. Further analyses were performed using this finalized model.

### Statistical analysis

Statistical analyses were performed using Python (version 3.9.18). Continuous variables are presented as mean ± standard deviation or median [min, max], as appropriate. Categorical variables are presented as counts and percentages. The Wilcoxon rank-sum test was used to compare continuous variables between independent groups (e.g., malignant *vs*. benign). The Chi-square test was used to compare categorical variables. ROC analysis was performed using scikit-learn python (v1.5.1). 95% confidence intervals (95% CI) for AUC, F1-score, sensitivity, specificity, and accuracy were calculated using a binomial distribution. All statistical tests were two-sided, with a significant level of *α* = 0.05, unless otherwise stated.

## Results

### Study population

**Figure 2:**
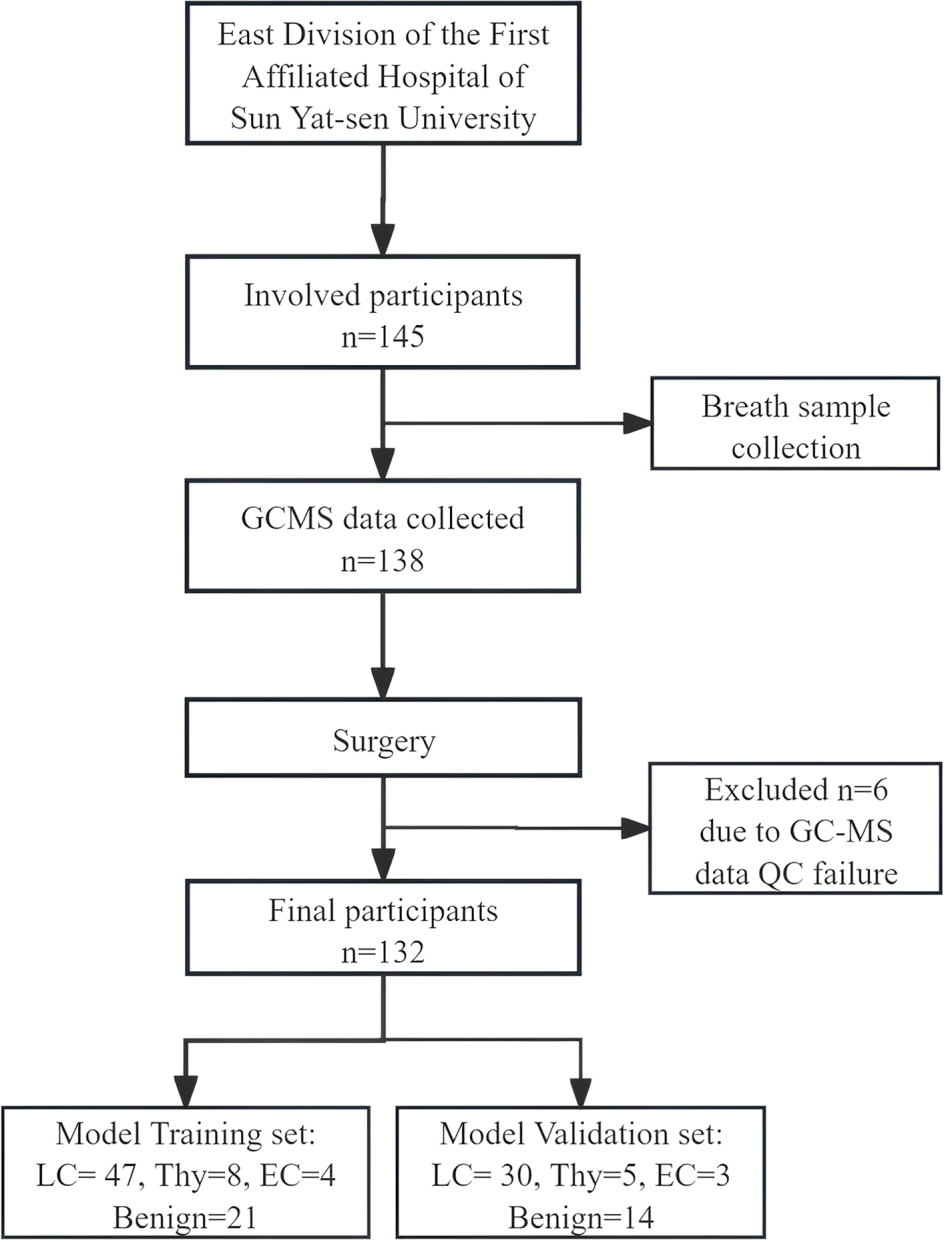
Schematic representations of the research framework.

145 participants were enrolled in this study. Exclusion criteria were applied to exclude individuals outside the age range of 18 to 80 years, those who declined participation, and those who provided invalid breath samples, resulting in a final cohort of 132 eligible participants for analysis. Among these, 77 were diagnosed with lung cancer, 13 with thymoma, 7 with esophageal cancer, and 35 had benign diseases, as confirmed by pathological results (**Figure 2**). The demographic and clinical data of these participants are presented in **Table 1**. Statistical comparisons between the case and control groups were conducted on basic demographic characteristics, including age, gender, body mass index (BMI), smoking and alcohol consumption status, and family cancer history. As detailed in **Table 1**, no significant difference was observed in these factors.

**Table 1:** Patient demographic and clinical characteristics.

Characteristics		Training set (*n* = 80)	Validation set (*n* = 52)	Total (*n* = 132)
Gender	Male	34 (42.50%)	18 (34.62%)	52 (39.39%)
	Female	46 (57.50%)	34 (65.38%)	80 (60.61%)
Age, year	Median [Min, Max]	58 [24, 81]	53 [13, 76]	57 [13, 81]
	Mean (SD)	56.58 (12.22)	52.48 (14.67)	54.92 (13.36)
BMI	Mean (SD)	23.9 (2.74)	22.49 (3.62)	23.28 (3.22)
Smoking	Never	40 (50.00%)	35 (67.31%)	75 (56.82%)
	Ever	4 (5.00%)	1 (1.92%)	5 (3.79%)
	Current	4 (5.00%)	2 (3.85%)	6 (4.55%)
	Unknown	32 (40.00%)	14 (26.92%)	46 (34.85%)
Drinking	Never	44 (55.00%)	38 (73.08%)	82 (62.12%)
	Ever	2 (2.50%)	0 (0.00%)	2 (1.52%)
	Current	2 (2.50%)	0 (0.00%)	2 (1.52%)
	Unknown	32 (40.00%)	14 (26.92%)	46 (34.85%)
Family cancer	Yes	2 (2.50%)	6 (11.54%)	8 (6.06%)
	No	45 (56.25%)	32 (61.54%)	77 (58.33%)
	Unknown	33 (41.25%)	14 (26.92%)	47 (35.61%)
Lesion size	< 10 mm	16 (20.00%)	9 (17.31%)	25 (18.94%)
	10–20 mm	25 (31.25%)	22 (42.31%)	47 (35.61%)
	20–30 mm	19 (23.75%)	7 (13.46%)	26 (19.70%)
	> 30 mm	17 (21.25%)	11 (21.15%)	28 (21.21%)
	Unknown	3 (3.75%)	3 (5.77%)	6 (4.55%)
Histopathology	Malignant	59 (73.75%)	38 (73.08%)	97 (73.48%)
	Benign	21 (26.25%)	14 (26.92%)	35 (26.51%)
Malignant subgroup	LC	47 (58.75%)	30 (57.69%)	77 (79.38%)
	Thymoma	8 (10.00%)	5 (9.62%)	13 (13.40%)
	EC	4 (5.00%)	3 (5.77%)	7 (7.22%)
Benign subgroup	Benign nodules	12 (15.00%)	8 (15.38%)	20 (57.14%)
	Benign others	9 (11.25%)	6 (11.54%)	15 (42.86%)
Benign histopathology	Hamartoma	2 (16.67%)	0 (0.00%)	2 (10.00%)
	Inflammation	5 (41.67%)	5 (62.50%)	10 (50.00%)
	Tuberculosis	1 (8.33%)	0 (0.00%)	1 (5.00%)
	Other nodule	4 (33.33%)	3 (37.50%)	7 (35.00%)
	Mediastinal cyst	2 (22.22%)	3 (60.00%)	5 (35.71%)
	Thymic hyperplasia	1 (11.11%)	0 (0.00%)	1 (7.15%)
	Thymolipoma	1 (11.11%)	0 (0.00%)	1 (7.15%)
	Esophageal hiatal hernia	0 (0.00%)	1 (20.00%)	1 (7.14%)
	Others	5 (55.56%)	1 (20.00%)	6 (42.86%)
LC histopathology	Adenocarcinoma	39 (82.98%)	27 (90.00%)	66 (85.71%)
	Squamous carcinoma	4 (8.51%)	2 (6.67%)	6 (7.79%)
	Others	4 (8.51%)	1 (3.33%)	5 (5.19%)
EC histopathology	Squamous carcinoma	4 (100%)	3 (100%)	7 (100%)
Thymoma histopathology	Type B2	6 (75.00%)	4 (80.00%)	10 (76.92%)
	Type B3	1 (12.5%)	1 (20.00%)	2 (15.38%)
	Type AB	1 (12.5%)	0 (0%)	1 (7.69%)
AJCC stages	Stage 0	2 (2.50%)	2 (3.85%)	4 (3.03%)
	Stage I	34 (42.5%)	23 (44.23%)	57 (43.18%)
	Stage II	10 (12.5%)	3 (5.77%)	13 (9.85%)
	Stage III	2 (2.50%)	5 (9.62%)	7 (5.30%)
	Stage IV	8 (10.00%)	5 (9.62%)	13 (9.85%)
	Unknown	24 (30.00%)	14 (26.92%)	38 (28.79%)

BMI, body mass index; EC, esophageal cancer; LC, lung cancer.

### VOC identification and feature selection

Initial statistical screening using the Wilcoxon rank-sum test and OPLS-DA revealed twenty-seven VOCs that exhibited differential abundance (*p* < 0.05) and high VIP scores (VIP > 1) when comparing exhaled breath samples from malignant and benign groups. These candidate VOCs then underwent compound identification and further refinement to exclude those associated with drug metabolism, environmental contaminants, or unrelated to the disease pathology. This rigorous filtering process ultimately yielded a final set of 18 potentially disease-relevant VOCs (**Supplementary Table S1**).

### Diagnostic model selection and feature optimization

**Figure 3:**
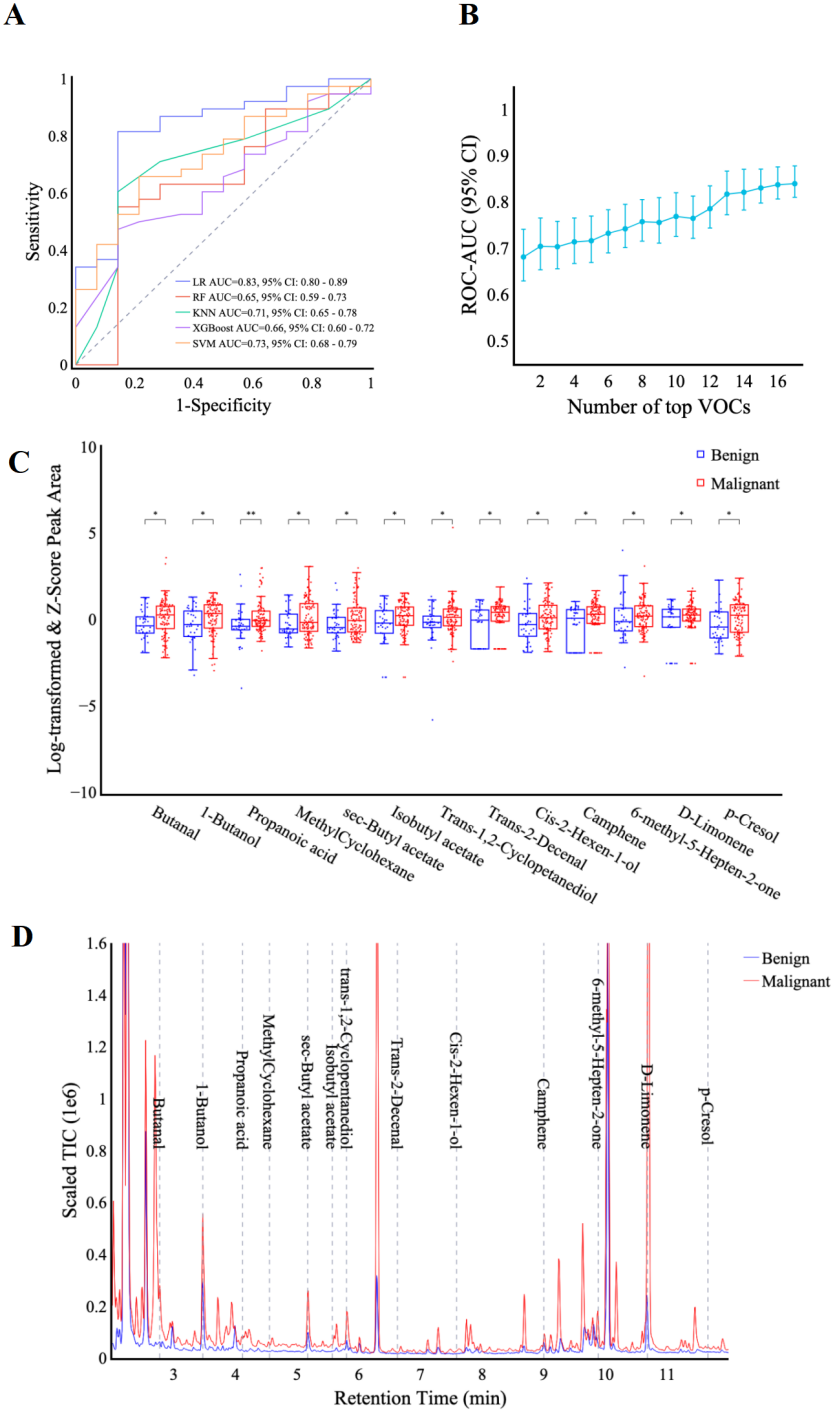
VOC identification and feature selection. **(A)** ROC curves display the classification performance of the VOC-models using five machine learning algorithms including LR, RF, KNN, XGBoost, and SVM. **(B)** the graph shows AUC values (y-axis) against the number of features (x-axis) in the VOC-model training set. **(C)** box plots comparing the scaled peak area of 13 VOCs in benign and malignant patients. y-axis: scaled VOC peak area by log-transformation and z-score normalization. Significance levels are denoted as follows: * p < 0.05, ** p < 0.01 (Rank-sum test). **(D)** representative chromatograph of 13 selected VOCs in malignant vs. benign patients. Scaled Total Ion Chromatogram TIC (Real TIC/1M).

To identify the optimal diagnostic model for differentiating benign from malignant thoracic lesions, five machine learning algorithms including logistic regression, SVM, random forest, KNN, and XGBoost were trained using the pre-selected panel of 18 VOCs. Comparison of the models revealed that logistic regression demonstrated robust performance in both the training and validation sets, achieving AUCs of 0.85 (95% CI: 0.82, 0.89) and 0.83 (0.80, 0.89), respectively (**Figure 3A**; **Supplementary Table S2**). The DeLong test indicated that logistic regression significantly outperformed the KNN, XGBoost, and SVM models in both datasets (p < 0.05). Furthermore, when compared to the random forest model, logistic regression demonstrated superior performance in the validation set (p < 0.01). Consequently, the logistic regression model was selected for further analysis and performance evaluation.

Final feature selection was conducted using the logistic regression algorithm to optimize model performance. Analysis of the AUC as a function of the number of top features revealed diminishing returns beyond 13 features. As incorporating additional features did not substantially improve the AUC, the top 13 features were selected for model development (**Figure 3B**). These identified compounds represent a diverse range of hydrocarbons, including methyl-cyclohexane, camphene, and d-limonene, as well as oxygenated species such as butanal, 1-butanol, propanoic acid, and *p*-cresol. **Table 2** provides a comprehensive list of these compounds and their corresponding discriminant values. Analysis of the scaled VOC peak area (**Figure 3C**) demonstrated that all 13 VOCs were present at elevated levels in the malignant group (*p* < 0.05). A representative chromatogram of the 13 VOCs in malignant and benign samples was shown in **Figure 3D**.

**Table 2:** 13 VOCs identified for model development.

VOC ID	Average retention time (min)	VOC name	*P* value	VIP	AUC	Literature reported associated with cancers	Possible biological origin(s)
70	2.738	Butanal	0.039	2.132	0.634	EC (46, 47, 49)	
114	3.498	1-Butanol	0.033	2.153	0.632	LC (31)	Alcohol metabolism, microbial fermentation
157	4.115	Propanoic acid	0.007	2.792	0.651	LC (32)	Lipid peroxidation, aldehyde metabolism
182	4.551	Methyl-cyclohexane	0.043	1.962	0.614	LC (33, 34), CRC (41)	Hydrocarbon metabolism, environmental exposure
206	5.115	Sec-Butyl acetate	0.036	1.774	0.602	LC (33)	Esterification of alcohol and acetic acid
226	5.57	Isobutyl acetate	0.018	2.804	0.622	Unknown	potential lipid peroxidation or microbial origin
242	5.779	Trans-1,2-Cyclopentanediol	0.034	2.04	0.612	Unknown	possibly microbial or environmental
309	6.626	Trans-2-Decenal	0.036	2.162	0.611	LC (39, 40)	Lipid peroxidation, oxidative stress
355	7.585	Cis-2-Hexen-1-ol, (Z)-	0.049	2.925	0.61	Unknown	Lipid peroxidation, plant-derived compounds
467	9	Camphene	0.025	2.259	0.611	LC (35)	Terpenoid metabolism
525	9.882	6-methyl-5-Hepten-2-one	0.049	1.9	0.584	GC (43–45), CRC (42), LC (37)	Fatty acid oxidation, isoprenoid metabolism
581	10.581	D-Limonene	0.039	1.473	0.612	LC (35, 36)	Terpene metabolism, dietary intake
663	11.661	p-Cresol	0.029	1.658	0.642	LC (38), EC/GC (50, 69–72)	Microbial metabolism (gut/oral), hepatic metabolism

AUC, area under curve; CRC, colorectal cancer; EC, esophageal cancer; GC, gastric cancer; LC, lung cancer; VOCs, volatile organic compounds; VIP, variable importance in the projection.

### Model performance in distinguishing benign from malignant thoracic lesions

**Figure 4:**
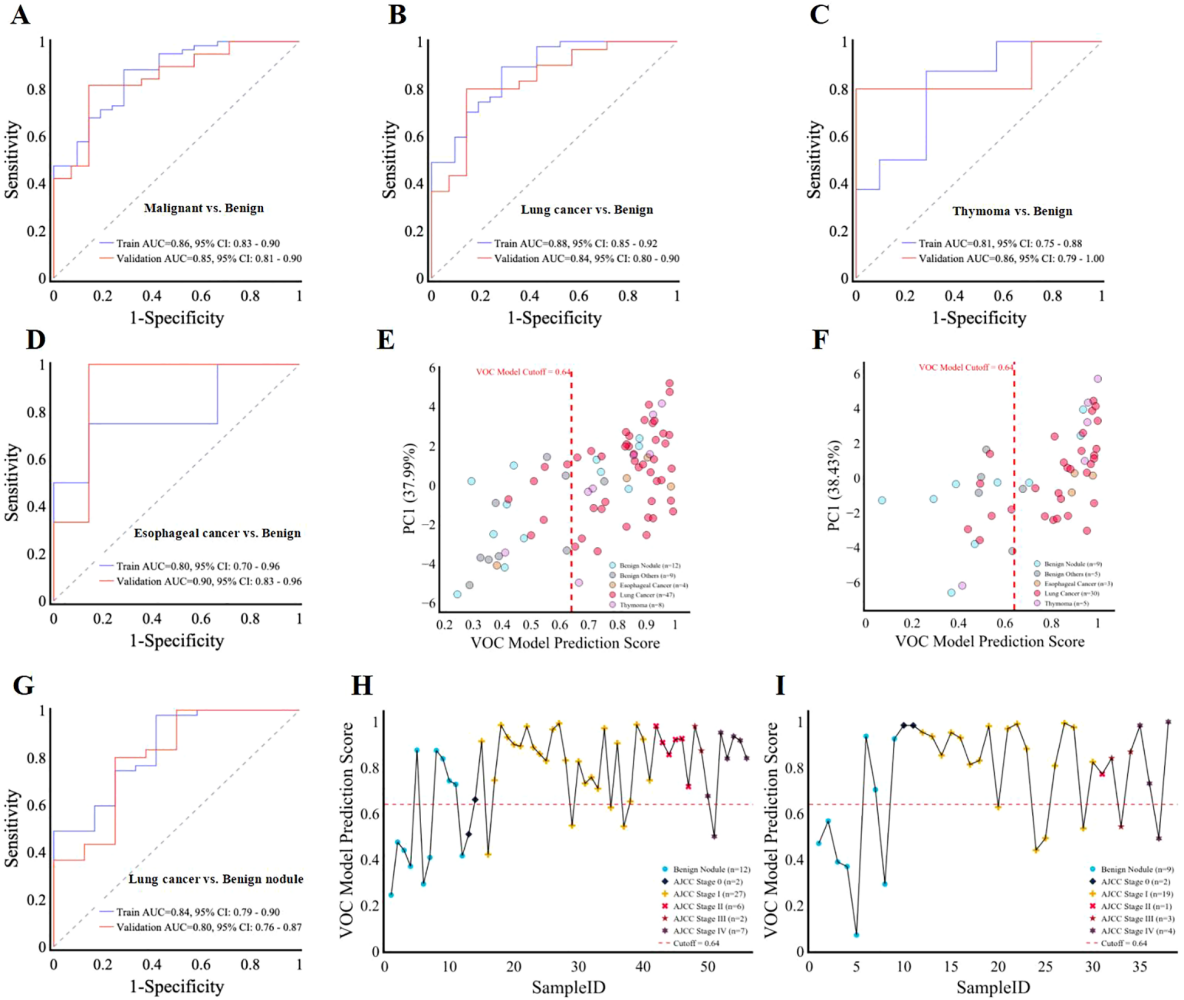
Model performance in distinguishing benign from malignant thoracic lesions. **(A–D, G)**, ROC curves for the 13-VOCs model in distinguishing malignant **(A)** *vs*. benign, lung cancer **(B)** *vs*. benign all, thymoma **(C)** *vs*. benign all, esophageal cancer **(D)** *vs*. benign all, and lung cancer **(G)** *vs*. benign nodules. **(E, F)**, Scatter plot depicting the relationship between VOC model prediction scores and the first principal component (PC1) in the training **(E)** and validation **(F)** sets, respectively. Each point represents an individual sample. The vertical red dashed line indicates the VOC model cutoff score of 0.64 used to discriminate between malignant and benign groups. **(H, I)**, VOC model prediction scores for individual samples (SampleID) across lung cancer and benign nodule groups in the training **(H)** and validation **(I)** sets, respectively. Samples are color-coded by category. The red dashed horizontal line represents the VOC model cutoff score of 0.64 used to distinguish between groups. Thymoma (n=13) and esophageal cancer (n=7) analyses are exploratory (limited sample size) and serve as hypothesis-generating observations.

In the training set (*n* = 80), the 13-VOC model demonstrated excellent performance with an AUC of 0.86 (0.83, 0.90), an accuracy of 0.83 (0.73, 0.89), a sensitivity of 0.86 (0.76, 0.93), and a specificity of 0.71 (0.50, 0.86). In the validation set (*n* = 52), the 13-VOC model achieved an AUC of 0.85 (0.81, 0.90), an accuracy of 0.79 (0.66, 0.88), a sensitivity of 0.82 (0.67, 0.91), and a specificity of 0.71 (0.45, 0.88), confirming its generalizability and clinical applicability (**Figure 4A**; **Supplementary Table S3**).

To further evaluate the performance of the detection model for individual cancer types, a subgroup analysis was conducted across various malignant thoracic lesions. Thymoma (n=13) and esophageal cancer (n=7) analyses are exploratory due to limited sample size and serve as hypothesis-generating observations. In the training set, the AUCs for lung cancer, thymoma, and esophageal cancer were 0.88 (0.85, 0.90), 0.81 (0.75, 0.88), and 0.80 (0.70, 0.96), respectively. In the validation set, corresponding AUCs were 0.84 (0.80, 0.90) for lung cancer, 0.86 (0.79, 1.00) for thymoma, and 0.91 (0.83, 0.95) for esophageal cancer (**Figures 4B–D**). To further visualize the model’s performance, prediction values for each participant were plotted against their actual disease status (lung cancer/thymoma/esophageal cancer *vs*. benign). Using a classification threshold of 0.64, the model achieved a high accuracy in the training set, correctly identifying 87.2% (75-94%) of lung cancer, 87.5% (53-98%) of thymoma, and 75% (30-95%) of esophageal cancer cases (**Figure 4E**). In the validation set, the model maintained high accuracy, correctly classifying 80% (63-91%) of lung cancer, 80% (38-96%) of thymoma, and 100% (44-100%) of esophageal cancer cases (**Figure 4F**). Additionally, the model demonstrated good specificity for benign lesions, correctly identifying 71.4% (50-86%, 45-88%) of such cases in both the training and validation sets (**Figures 4E–F**). These findings emphasize the model’s robust performance and generalized applicability in detecting various malignant thoracic lesions. Importantly, its ability to differentiate benign lesions underscores its potential to minimize unnecessary interventions and overtreatment, supporting its use in clinical practice.

### Model performance in differentiating pulmonary lesions and across different lung cancer AJCC stages

Building upon previous findings, we further investigated the model’s ability to differentiate malignant and benign pulmonary lesions. In the training set (*n* = 59), the 13-VOC model achieved an AUC of 0.82 (0.68, 0.95), sensitivity of 0.89 (0.77, 0.95), and specificity of 0.58 (0.32, 0.81). In the validation set (*n* = 38), the model exhibited an AUC of 0.79 (0.57, 0.98), sensitivity of 0.80 (0.63, 0.91), and specificity of 0.63 (0.31, 0.86) (**Figure 4G**; **Supplementary Table S3**).

Early detection of lung cancer is critical in clinical practice, allowing for timely interventions and curative resections that substantially increase patient survival rates. To assess our model’s efficacy in diagnosing early lung cancer, we used the model to differentiate between various lung cancer stages and benign nodules. The predictive performance of the model was graphically demonstrated by plotting individual participant predictions against their corresponding ground truth classifications (lung cancer stages or benign nodule). With a predetermined classification cut-off at 0.64, the 13-VOC model demonstrated strong performance in identifying early-stage lung cancer, achieving high accuracy for stage 0 + I + II lung cancer (85.7% [70.6-93.7%]) and stage III + IV lung cancer (88.9% [56.5-98%]) in the training set, though the accuracy for benign nodules was comparatively lower at 58.3% (32-80.7%) (**Figure 4H**). In the validation set, the model maintained robust performance for stage 0 + I + II lung cancer (81.8% [61.5-92.7%]) and improved accuracy for benign nodules (66.7% [35.4-87.9%]), though there was a slight decrease in accuracy for stage III+IV lung cancer (71.4% [35.9-91.8%]) (**Figure 4I**). These findings highlight the model’s potential for early detection and timely treatment of lung cancer, as well as its capacity to reduce unnecessary interventions and overtreatment—an essential consideration in clinical decision-making. However, further optimization is necessary to enhance its ability to accurately differentiate benign nodules and address variability in diagnosing advanced-stage lung cancer.

### Comparison of the diagnostic performance of the VOC model with traditional methods using serum tumor biomarkers

**Figure 5:**
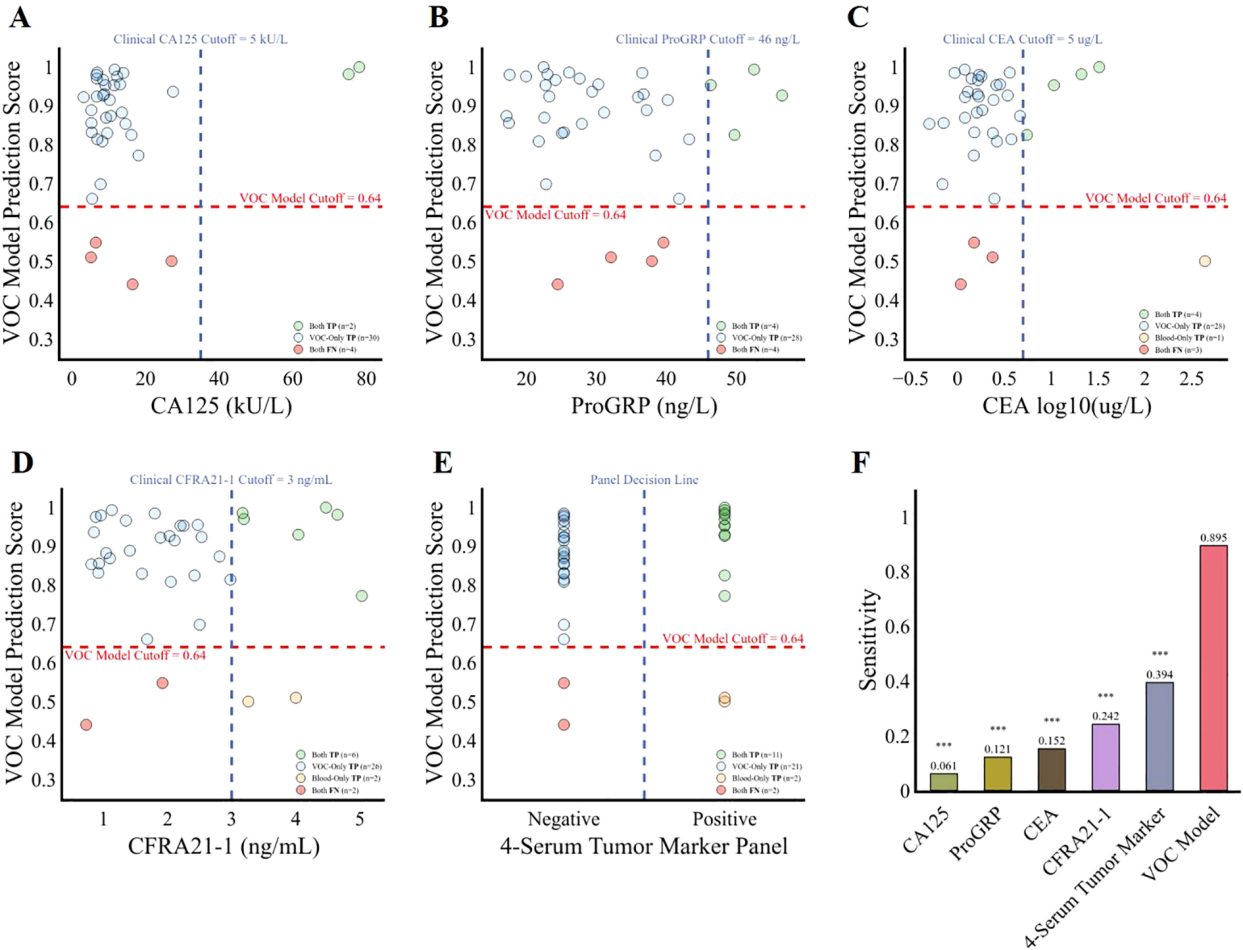
Comparison of the diagnostic performance of the 13-VOCs model with clinical serum tumor biomarkers among lung cancer patients. **(A–E)**, diagnostic prediction of the lung cancer patients using the 13-VOCs model and clinical markers CA125, ProGRP, CEA, CFRA21-1, and the 4-serum tumor marker panel respectively. The blue vertical dashed line represents the clinical serum tumor biomarkers at various cutoffs, while the red horizontal dashed line indicates the VOC model cutoff score at 0.64. Each point corresponds to an individual lung cancer sample (n=36). **(F)**, comparison of the four individual serum tumor marker, 4-serum tumor marker panel and 13-VOCs models’ detection sensitivity in predicting lung cancer patients. Sensitivity differences between the 13-VOC model, each individual serum tumor marker, and the 4-serum tumor marker panel were evaluated using McNemar’s test. Significance is denoted as follows: ***, p < 0.001.

To determine whether the model represents an advancement in tumor diagnosis, we compared its predictive accuracy against that of four established clinical tumor biomarkers: CA125, ProGRP, CEA, and CFRA21-1. Among the 36 lung cancers patients, the discriminative sensitivities of CA125, ProGRP, CEA, and CFRA21–1 were 0.061, 0.121, 0.152 and 0.242 respectively, while the 13-VOCs model showed a paired discriminative sensitivity of 0.895 (*p* < 0.001) (**Figures 5A–D, F**). Given the common clinical practice of combining these four biomarkers to enhance specificity, we hypothesized that classifying individuals as positive for lung cancer if any of the serum tumor biomarkers fell outside the normal range (i.e., CA125: 0–35 KU/L, ProGRP: 0–46 ng/L, CEA: 0-5μg/L, CFRA21-1: 0–3 ng/L) could yield improved sensitivity. Of particular note, our 13-VOCs model significantly outperformed the 4-serum tumor marker panel, achieving a sensitivity of 0.895 compared to 0.394 (*p* < 0.001) (**Figures 5E, F**). Importantly, this superior performance was not attributable to an elevated false positive rate (**Figure 4**). These findings suggested that the 13-VOCs model represents a more robust diagnostic tool, potentially offering particular advantages for early detection, an area where traditional serum biomarkers have limited utility.

### Model performance for postoperative monitoring and follow-up

**Figure 6:**
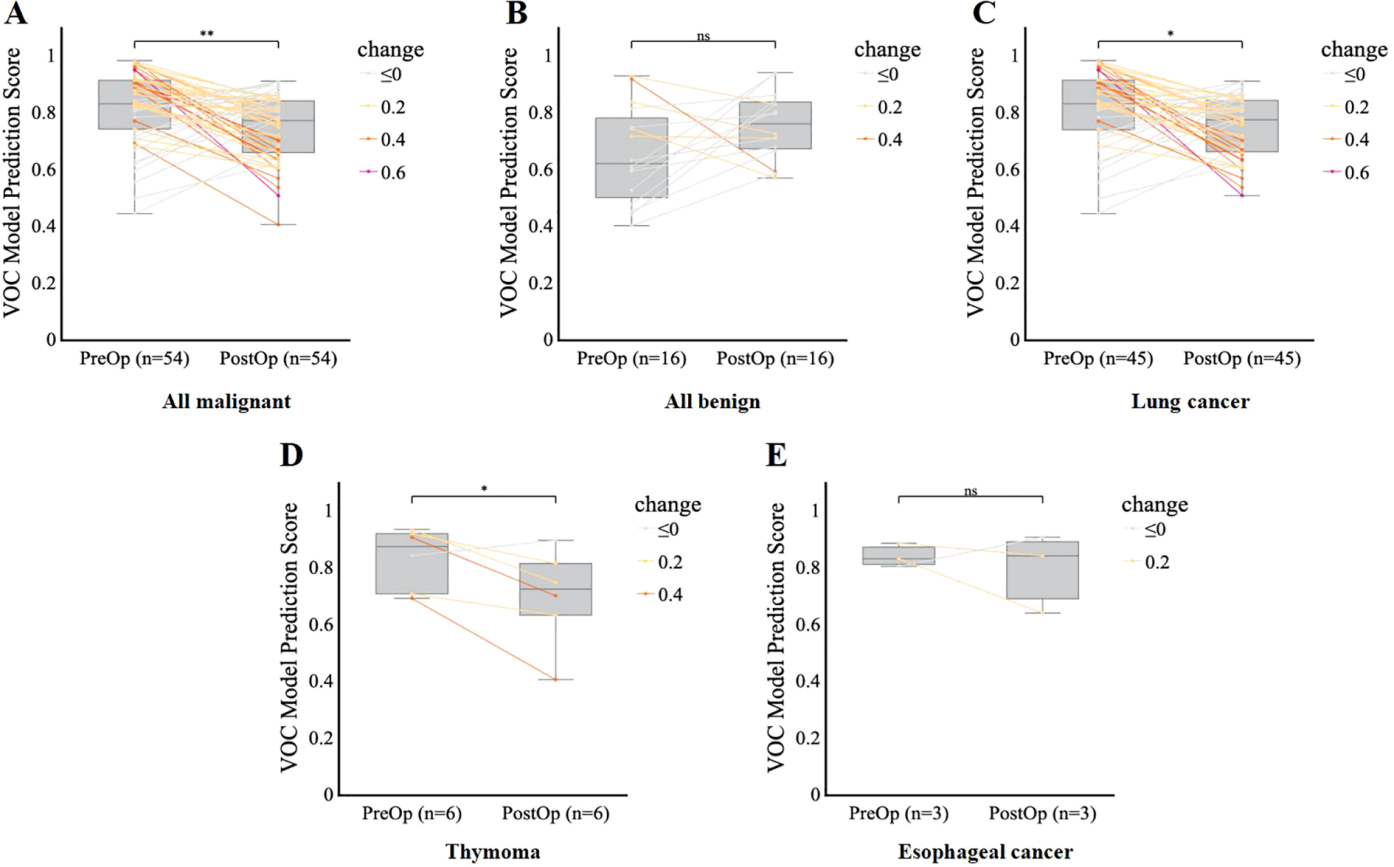
Model performance for postoperative monitoring and follow-up. **(A–E)**, Comparison of VOC model prediction scores before (PreOp) and after (PostOp) surgery in the overall malignant group **(A)**, benign group **(B)**, lung cancer group **(C)**, thymoma group **(D)**, and esophageal cancer group **(E)**, respectively. Each line represents an individual patient’s change in score, color-coded by the magnitude of change. Boxplots show the median and interquartile range. Comparisons in VOC prediction by scores postoperatively were performed using a paired t-test. Significance is denoted as follows: *p < 0.05, **p < 0.01, ns = not significant (p ≥ 0.05).

To assess whether the model accurately reflects dynamic changes in disease status and further validate that the features it captures are closely associated with disease activity or burden, we analyzed and compared the model’s score changes between preoperative assessments and postoperative timepoints, 7 days to 1 month after surgery. Among the cancer patients (*n* = 54), postoperative predicted probabilities were significantly lower than preoperative probabilities (*p* < 0.01), indicating a measurable decrease in predicted disease burden following surgical intervention (**Figure 6A**). Subgroup analysis confirmed this trend in both lung cancer (*p* < 0.05) (**Figure 6C**) and thymoma (*p* < 0.05) (**Figure 6D**), with postoperative scores remaining consistently lower across these malignancies. The postoperative reduction in esophageal cancer was not statistically significant (*p* > 0.05) (**Figure 6E**), possibly due to the limited sample size. Notably, there was no significant difference in predicted probabilities between the postoperative and preoperative groups in cases of benign disease (*p* > 0.05) (**Figure 6B**). Collectively, these findings demonstrate that the model effectively reflects the reduction in disease burden following lung cancer surgery, highlighting its potential utility for assessing the completeness of resection and detecting early signs of postoperative recurrence.

## Discussion

This study aimed to develop and validate a novel machine learning model for the early diagnosis of thoracic malignancies using exhaled VOCs as biomarkers. To our knowledge, our findings demonstrate for the first time the feasibility of employing a single panel of VOC profiles to differentiate between benign and malignant thoracic lesions, particularly lung, esophageal, and thymic tumors.

To determine the optimal classifier for metabolomics data analysis, we evaluated five machine learning algorithms: logistic regression, random forest, k-nearest neighbor, XGBoost, and support vector machine, on training and validation datasets. Logistic regression demonstrated robust performance on both sets, making it the ideal choice for the baseline model. While algorithms like Random Forest and XGBoost showed some promise, they were not pursued due to their increased complexity and computational demands, without significant improvement in testing set performance. Given its simplicity, interpretability, efficiency, and strong generalization, logistic regression emerged as the optimal model for metabolomics data analysis. The 13-VOC model constructed by logistic regression algorithm achieved high accuracy in classifying thoracic tumors, with an AUC of 0.85, sensitivity of 82%, and specificity of 71%, representing a clinically significant advancement over existing clinical markers, which only achieved a sensitivity of 39.4%. Notably, the model exhibited robust performance in distinguishing early-stage lung cancer, suggesting its potential as a non-invasive screening tool.

Thirteen VOCs were identified as potential biomarkers for distinguishing malignant from benign thoracic lesions, many of which have established or emerging links to various cancer metabolism and pathogenesis. Several VOCs, including 1-butanol (31), propanoic acid (32), methyl-Cyclohexane (33, 34), sec-Butyl acetate (33), camphene (35), D-Limonene (35, 36), 6-methyl-5-Hepten-2-one (37), and *p*-cresol (38), have been previously reported as elevated in lung cancer and other malignancies.

Emerging evidence suggests that these VOCs may reflect key metabolic alterations characteristic of cancer pathogenesis. Trans-2-Decenal (39, 40), an alkenal mutagen found in cooking oil fumes, has been shown to promote oxidative DNA damage through reactive oxygen species formation, a well-established mechanism implicated in lung carcinogenesis, suggesting increased risk for individuals with frequent exposure (39). Methyl-cyclohexane, which has also been implicated in distinguishing colorectal cancer from healthy controls, may indicate broader metabolic reprogramming in malignancy (41). 6-methyl-5-Hepten-2-one, potentially linked to increased fatty acid oxidation (42), a hallmark of cancer cell metabolism, was reported to be elevated in various gastrointestinal cancers, including colorectal (42) and gastric cancer (43–45).

Butanal, elevated in esophagogastric cancer (46), may accumulate due to genetic dysregulation of its metabolic pathways or as a byproduct of lipid peroxidation—a process often amplified by chronic inflammation in the tumor microenvironment. This aligns with the recognized role of oxidative stress in cancer progression (47, 48). Furthermore, alterations in the gut microbiome, frequently observed in esophageal cancer, can modulate butanal production and metabolism (49), highlighting the interplay between host metabolism and microbial communities in cancer.

*p*-Cresol, with its complex metabolism influenced by gut and oral microbiota, hepatic processes, and disease state, has been identified as a potential breath biomarker in various cancers, including esophageal, gastric, thyroid, breast, oral, and lung cancers, and even in some non-malignant conditions (50, 51). This broad association suggests *p*-cresol and other VOCs may serve as general indicators of metabolic dysregulation or malignancy.

In contrast, isobutyl acetate, trans-1,2-Cyclopentanediol, and cis-2-Hexen-1-ol currently lack well-established links to cancer pathogenesis. Isobutyl acetate has been primarily reported as a marker for microbial (specifically Candida albicans) activity, particularly in respiratory infections (52). It may also indirectly contribute to metabolic disorders like obesity and diabetes through oxidative stress and neuroinflammation (53), and potentially to cardiovascular disease via ROS-mediated metabolic dysregulation (54). Further studies should include metabolomic and pathway enrichment analyses, such as KEGG, to elucidate their metabolic origins and explore potential sources like lipid peroxidation or microbial dysbiosis.

The observation that several VOCs are associated with multiple cancer types suggests they may serve as general indicators of malignancy or reflect shared metabolic pathways. Combining these VOCs into a diagnostic model is justified by their diverse origins and links to various cancer-related pathways, including genetic dysregulation, oxidative stress, lipid peroxidation, and microbiome alterations, enabling the capture of a more comprehensive metabolic fingerprint of thoracic malignancies, potentially improving diagnostic accuracy. Nevertheless, further mechanistic studies are needed to elucidate how these VOCs specifically relate to cancer pathogenesis and to validate their clinical utility as biomarkers.

Furthermore, the model’s ability to track changes in VOC profiles over time, as demonstrated by the significant decrease in predicted risk following surgery, highlights its potential for monitoring disease progression and treatment response. Wang et al. demonstrated the feasibility of using perioperative dynamic breathomics to identify a panel of VOCs as potential biomarkers for lung cancer (55). By comparing VOC profiles before and after surgery, they identified 16 VOCs that were significantly altered in lung cancer patients, and a machine learning model based on these VOCs achieved high accuracy of 86.9% in differentiating between lung cancer patients and healthy controls. Nardi-Agmon et al. explored the potential of breath analysis for monitoring the response to anticancer treatment in patients with advanced lung cancer (56). By utilizing a panel of three VOCs identified as significant indicators of treatment outcomes, this approach may provide a rapid and non-invasive method for assessing treatment response, potentially enabling earlier detection of treatment failure compared to conventional imaging techniques. These findings highlight the growing evidences supporting breath analysis as a valuable tool for lung cancer management, with the ability to detect dynamic changes in VOC profiles pre- and post-treatment suggesting its potential as a complementary approach to existing diagnostic and monitoring strategies.

However, several limitations of this study should be acknowledged. Firstly, although our cohort was prospectively enrolled, the sample size (n=132) and subtype distribution (lung cancer 79.4%, thymoma 13.4%, esophageal cancer 7.2%) reflect the underlying epidemiology of thoracic malignancies (1, 3, 4). This distribution enabled robust differentiation between malignant and benign lesions, but the small numbers for rarer subtypes limit the strength of conclusions for thymoma and esophageal cancer. These analyses are exploratory and serve as preliminary, hypothesis-generating observations. External validation in larger, multi-center cohorts, particularly through collaboration with international consortia for rare thoracic tumors, is essential to confirm these findings and support broader clinical application. Secondly, the reliance on GC-MS for VOC analysis presents challenges for clinical implementation. GC-MS is a complex, time-consuming, and expensive technique that requires specialized equipment and expertise, making it less feasible for routine clinical use (57). To address this, future research should focus on validating these findings using point-of-care testing (POCT) devices, such as micro-GC systems (58–65), electronic noses (66), or wearable VOC sensors (67), which offer real-time, bedside breath analysis for rapid clinical decision-making. However, challenges remain, including achieving sufficient sensor sensitivity and selectivity, minimizing sensor drift and environmental interference, and standardizing protocols. Progress in materials science and AI-driven data analysis, along with interdisciplinary collaboration, will be crucial to address these issues. Pilot studies in clinical settings are also needed to assess practicality, cost-effectiveness, and user acceptance, ultimately supporting the adoption of breath analysis in routine healthcare. Large-scale validation studies using such technologies could pave the way for the widespread adoption of breath analysis in clinical practice (58–63). Thirdly, this study focused only on lung cancer, thymoma, and esophageal cancer, limiting its applicability to other thoracic malignancies. Future research should include mesothelioma, mediastinal tumors, and other rare thoracic cancers to develop a more comprehensive model, which could improve breath analysis for diagnosis and monitoring across the entire spectrum of thoracic oncology. Fourthly, while the study demonstrates the potential of VOC analysis, further research is needed to elucidate the underlying biological mechanisms and to address the technical challenges associated with breath sample collection and analysis. Finally, although LDCT is widely used for lung cancer screening, it carries high costs and a notable false-positive rate, which can lead to unnecessary follow-up tests and increased patient anxiety (68). In contrast, breath-based VOC analysis offers a non-invasive, radiation-free, and potentially more cost-effective screening approach. However, our current methodology relies on GC-MS, which is not yet feasible for large-scale screening due to its expense and complexity. The development of portable, point-of-care VOC detection platforms may help overcome these limitations, enabling broader clinical implementation and possibly reducing the economic and logistical burden associated with current screening methods. Future studies should directly compare the clinical and economic outcomes of VOC-based POCT and existing modalities such as LDCT to determine the most effective and sustainable strategies for early cancer detection. Despite these limitations, this study provides a strong foundation for the development of breath analysis as a valuable tool for the early detection, diagnosis, and monitoring of thoracic cancers.

## Conclusion

This study establishes the effectiveness of a breath-derived VOC model in distinguishing malignant and benign thoracic lesions, demonstrating its capability for multi-cancer detection and early-stage diagnosis. By pioneering breathomics for simultaneous identification of multiple thoracic malignancies and exploring its potential for postoperative monitoring, this work introduces a novel integration of non-invasive diagnostics with therapeutic surveillance. Compared to traditional serum biomarkers, the approach demonstrates superior sensitivity while eliminating invasive sampling, offering a patient-friendly alternative with clinical scalability. The methodology holds promise for improving early cancer detection and real-time postoperative evaluation, potentially enhancing clinical decision-making and personalized patient management. Future efforts should prioritize validation in broader populations, refinement of the predictive model, and development of point-of-care devices to facilitate clinical translation and improve patient outcomes.

## Data Availability

The raw data supporting the conclusions of this article will be made available by the authors, without undue reservation.
