# Supplementary material for: Machine learning assisted breathomic approach for early-stage thoracic cancer detection
Source: Front Oncol. 2025 Sep 17;15:1635280. doi: 10.3389/fonc.2025.1635280 (PMC12483886; doi:10.3389/fonc.2025.1635280)
Supplement: Supplementary file 1 [file DataSheet1.docx]

**Supplementary information**

**Machine Learning Assisted Breathomic Approach for Early-Stage Thoracic Cancer Detection**

**Materials and Methods**

TD-GC-MS analytical conditions

The injector temperature was 300°C. Nitrogen carrier gas was used at a flow rate of 1 mL/min. The thermal desorption unit was operated under standard conditions. The flow path temperature was maintained at 200 °C, with a standby split flow of 10 mL/min. Prior to desorption, the sample tube was pre-purged at a flow rate of 10 mL/min for 1 min to remove potential moisture. Primary desorption was performed at 320°C for 5 min, with cold trap and split flow rates set at 50 mL/min and 10 mL/min, respectively. The cold trap temperature was initially maintained at -5 °C and then ramped to 320 °C over 3 min. Subsequently, the cold trap was purged at 50 mL/min for 1 min, with a split flow of 10 mL/min, ensuring efficient transfer of analytes to the GC-MS system.

The GC-MS system was operated under the following conditions. A capillary column (HP-5MS, 30 m × 0.25 mm × 0.25 μm) was used for compound separation. Nitrogen was used as the carrier gas, with a constant flow rate of 1.0 mL/min. The oven temperature program was as follows: an initial temperature of 30 °C was held for 10 min, then increased at a rate of 8 °C/min to 90 °C and held for 7 min. The transfer line and ionization source temperatures were set at 280°C, while the quadrupole temperature was maintained at 150 °C.

**Table S1.** The 27 preliminarily selected VOCs with different levels between benign and malignant groups.

| NO. | Alignment ID | Average Rt (min) | VOC name | Note |
| --- | --- | --- | --- | --- |
| 1 | 51 | 2.494 | Methanesulfonyl chloride | environmental contaminants |
| 2 | 65 | 2.648 | Cyclopentane |  |
| 3 | 70 | 2.738 | Butanal |  |
| 4 | 114 | 3.498 | 1-Butanol |  |
| 5 | 146 | 4.014 | 1,3,6-Trioxocane | environmental contaminants |
| 6 | 157 | 4.115 | Propanoic acid |  |
| 7 | 182 | 4.551 | Cyclohexane, methyl- |  |
| 8 | 206 | 5.115 | sec-Butyl acetate |  |
| 9 | 226 | 5.57 | Isobutyl acetate |  |
| 10 | 227 | 5.575 | 4-Fluorohistamine | drug metabolism |
| 11 | 242 | 5.779 | 1,2-Cyclopentanediol, trans- |  |
| 12 | 309 | 6.626 | 2-Decenal, (E)- |  |
| 13 | 355 | 7.585 | 2-Hexen-1-ol, (Z)- |  |
| 14 | 435 | 8.497 | cycloterasiloxane,octamethyl- | environmental contaminants |
| 15 | 467 | 9 | Camphene |  |
| 16 | 525 | 9.882 | 5-Hepten-2-one, 6-methyl- |  |
| 17 | 581 | 10.581 | D-Limonene |  |
| 18 | 663 | 11.661 | p-Cresol |  |
| 19 | 930 | 14.894 | 3-Trifluoroacetoxydodecane | environmental contaminants |
| 20 | 995 | 15.675 | Indole |  |
| 21 | 1021 | 16.102 | 7-Hexadecenal, (Z)- |  |
| 22 | 1292 | 19.479 | 2-Trifluoroacetoxypentadecane | environmental contaminants or drug metabolism |
| 23 | 1470 | 21.952 | Tertbutyloxyformamide, N-methyl-N-[4-(1-pyrrolidinyl)-2-butynyl]- | drug metabolism |
| 24 | 1486 | 22.181 | Spiro-1-(cyclohex-2-ene)-2'-(5'-oxabicyclo[2.1.0]pentane), 1',4',2,6,6-pentamethyl- | unrelated to disease |
| 25 | 1528 | 22.762 | 4-Fluoro-1-methyl-5-carboxylic acid, ethyl(ester) | drug metabolism |
| 26 | 1745 | 25.649 | Pterin-6-carboxylic acid |  |
| 27 | 1886 | 27.564 | Octadecanoic acid |  |

**Table S2**. Model performances in five machine learning algorithms

| Algorithm | Dataset | ROC-AUC (95% CI) | Logistic Regression vs. Other model  *p* value |
| --- | --- | --- | --- |
| Logistic Regression | Train Set (n=80) | 0.85 (0.82, 0.89) | NA |
|  | Validation Set (n=52) | 0.83 (0.80, 0.89) | NA |
| Random Forest | Train Set (n=80) | 0.89 (0.88, 0.93) | 0.19 |
|  | Validation Set (n=52) | 0.65 (0.59, 0.73) | <0.01 |
| KNN | Train Set (n=80) | 0.73 (0.69, 0.79) | <0.01 |
|  | Validation Set (n=52) | 0.71 (0.65, 0.78) | <0.01 |
| XGBoost | Train Set (n=80) | 0.92 (0.90, 0.94) | 0.01 |
|  | Validation Set (n=52) | 0.66 (0.60, 0.72) | <0.01 |
| SVM | Train Set (n=80) | 0.79 (0.75, 0.84) | 0.04 |
|  | Validation Set (n=52) | 0.73 (0.68, 0.79) | 0.01 |

**Table S3**. Model performances in five machine learning algorithms

| Models | Datasets | ROC-AUC | F1 | Accuracy | Sensitivity | Specificity |
| --- | --- | --- | --- | --- | --- | --- |
| Malignant vs. benign | Train Set (n=80) | 0.86 (0.83, 0.90) | 0.88 | 0.83 (0.73, 0.89) | 0.86 (0.76, 0.93) | 0.71 (0.50, 0.86) |
|  | Validation Set (n=52) | 0.85 (0.81, 0.90) | 0.85 | 0.79 (0.66, 0.88) | 0.82 (0.67, 0.91) | 0.71 (0.45, 0.88) |
| LC vs. Benign | Train Set (n=68) | 0.88 (0.85, 0.92) | 0.87 | 0.82 (0.72, 0.90) | 0.87 (0.75, 0.94) | 0.71 (0.50, 0.86) |
|  | Validation Set (n=44) | 0.84 (0.80, 0.90) | 0.83 | 0.77 (0.63, 0.87) | 0.80 (0.63, 0.91) | 0.71 (0.45, 0.88) |
| Thymoma vs. Benign | Train Set (n=29) | 0.81 (0.75, 0.88) | 0.67 | 0.76 (0.58, 0.88) | 0.88 (0.53, 0.98) | 0.71 (0.50, 0.86) |
|  | Validation Set (n=25) | 0.86 (0.79, 1.00) | 0.62 | 0.74 (0.51, 0.88) | 0.80 (0.38, 0.96) | 0.71 (0.45, 0.88) |
| EC vs. Benign | Train Set (n=19) | 0.80 (0.70, 0.96) | 0.46 | 0.72 (0.52, 0.86) | 0.75 (0.30, 0.95) | 0.71 (0.50, 0.86) |
|  | Validation Set (n=17) | 0.91 (0.83, 0.96) | 0.6 | 0.77 (0.53, 0.90) | 1.00 (0.44, 1.00) | 0.71 (0.45, 0.88) |
| LC vs. Benign nodules | Train Set (n=59) | 0.82 (0.68, 0.95) | 0.89 | 0.83 (0.72, 0.91) | 0.89 (0.77, 0.95) | 0.58 (0.32, 0.81) |
|  | Validation Set (n=39) | 0.79 (0.57, 0.98) | 0.84 | 0.76 (0.61, 0.87) | 0.80 (0.63, 0.91) | 0.63 (0.31, 0.86) |

Note: Thymoma and EC analyses are exploratory due to sample size constraints (n<15)
